# Noninvasive Delineation of Glioma Infiltration with Combined 7T Chemical Exchange Saturation Transfer Imaging and MR Spectroscopy: A Diagnostic Accuracy Study

**DOI:** 10.3390/metabo12100901

**Published:** 2022-09-24

**Authors:** Yifan Yuan, Yang Yu, Yu Guo, Yinghua Chu, Jun Chang, Yicheng Hsu, Patrick Alexander Liebig, Ji Xiong, Wenwen Yu, Danyang Feng, Baofeng Yang, Liang Chen, He Wang, Qi Yue, Ying Mao

**Affiliations:** 1 Department of Neurosurgery, Huashan Hospital, Fudan University, Shanghai 200040, China; 2 National Center for Neurological Disorders, Shanghai 201112, China; 3 Shanghai Key Laboratory of Brain Function and Restoration and Neural Regeneration, Fudan University, Shanghai 200032, China; 4 Department of Radiology, Huashan Hospital, Fudan University, Shanghai 200040, China; 5 MR Collaboration, Siemens Healthineers Ltd., Shanghai 310000, China; 6 Siemens Healthcare GmbH, Henkestr. 127, 91052 Erlangen, Germany; 7 Department of Pathology, Huashan Hospital, Fudan University, Shanghai 200040, China; 8 Institute of Science and Technology for Brain-Inspired Intelligence, Fudan University, Shanghai 200433, China; 9 Human Phenome Institute, Fudan University, Shanghai 200433, China; 10 Key Laboratory of Computational Neuroscience and Brain-Inspired Intelligence (Fudan University), Ministry of Education, Shanghai 200433, China

**Keywords:** CEST, MRS, FET-PET, ultra-high field MRI, glioma

## Abstract

For precise delineation of glioma extent, amino acid PET is superior to conventional MR imaging. Since metabolic MR sequences such as chemical exchange saturation transfer (CEST) imaging and MR spectroscopy (MRS) were developed, we aimed to evaluate the diagnostic accuracy of combined CEST and MRS to predict glioma infiltration. Eighteen glioma patients of different tumor grades were enrolled in this study; ^18^F-fluoroethyltyrosine (FET)-PET, amide proton transfer CEST at 7 Tesla(T), MRS and conventional MR at 3T were conducted preoperatively. Multi modalities and their association were evaluated using Pearson correlation analysis patient-wise and voxel-wise. Both CEST (R = 0.736, *p* < 0.001) and MRS (R = 0.495, *p* = 0.037) correlated with FET-PET, while the correlation between CEST and MRS was weaker. In subgroup analysis, APT values were significantly higher in high grade glioma (3.923 ± 1.239) and IDH wildtype group (3.932 ± 1.264) than low grade glioma (3.317 ± 0.868, *p* < 0.001) or IDH mutant group (3.358 ± 0.847, *p* < 0.001). Using high FET uptake as the standard, the CEST/MRS combination (AUC, 95% CI: 0.910, 0.907–0.913) predicted tumor infiltration better than CEST (0.812, 0.808–0.815) or MRS (0.888, 0.885–0.891) alone, consistent with contrast-enhancing and T2-hyperintense areas. Probability maps of tumor presence constructed from the CEST/MRS combination were preliminarily verified by multi-region biopsies. The combination of 7T CEST/MRS might serve as a promising non-radioactive alternative to delineate glioma infiltration, thus reshaping the guidance for tumor resection and irradiation.

## 1. Introduction

Glioma is the most malignant primary brain tumor with a prevalence of 4–5/100,000 and is also a leading cause of cancer mortality in adults [1]. Surgery serves as the first-line treatment to debulk tumors and obtain tissues for pathologic analysis [2]. A broad consensus agrees that the extent of tumor removal is positively correlated with patient survival and residual tumor always leads to early recurrence [3]. Therefore, precise delineation of tumor infiltration guides clinical decision-making for glioma therapy.

MRI, the most widely used technique for glioma diagnosis, has been applied in intraoperative neuro-navigation and irradiation guidance. However, since blood–brain barrier (BBB) remains intact in some tumor regions regardless of glioma grade, the enhanced signal on post-contrast T1 images is not precise enough to delineate tumor infiltration [4]. Moreover, discriminating tumor infiltration from peri-tumor edema on T2 images is also challenging. An amino acid PET scan uses radio-labeled tracers, such as 18F-Fluoroethyltyrosine (FET), for tumor imaging according to hyper-metabolism in the tumor microenvironment has been recommended by the European Association for Neuro-Oncology (EANO) as the optimal technique for identification of glioma infiltration [5]. However, drawbacks such as radiation exposure and high cost limit its application [6].

Novel modality development, as well as ultra-high-field application, has paved the way for potential MR alternatives of amino acid PET. Chemical exchange saturation transfer (CEST), based on mobile proton exchange among various molecules, can generate specific MR contrast at different radiofrequency pulses, thus detecting endogenous proteins and acidosis [7]. Amide Proton Transfer (APT), a standard CEST approach that targets amide protons and provides high contrast at a frequency of 3.5 ppm, has been exploited to assist in glioma diagnosis due to elevated mobile peptides and proteins in malignant tumors [8,9,10]. Recently ultra-high-field has also been introduced for the CEST approach and demonstrated better performance for glioma diagnosis [11]. MRS is another modality that measures a variety of biochemicals in tumors and has been used clinically to quantify metabolite concentrations in glioma. The Cho/NAA Ratio (CNR), calculated from MRS peaks, positively predicts tumor malignancy and has been used for guidance intraoperatively to metabolically detect tumor boundaries [12]. Considering that every modality has inherent drawbacks, multimodal approaches combining two or more MR modalities have emerged as new methods to provide high sensitivity for tumor diagnosis [13]. Although both methods reflect tumor metabolism, few studies have combined CEST with MRS in the treatment of gliomas.

In this study, we first explored correlations between CEST and MRS and then chose FET-PET as the reference to investigate applying multimodal MR imaging for metabolic glioma evaluation and tumor extent delineation. We aimed to develop a novel noninvasive diagnostic method for glioma, thus assisting precise resection and irradiation.

## 2. Materials and Methods

### 2.1. Study Population

Study participants were prospectively recruited from patients diagnosed with glioma preoperatively by experienced radiologists and who had received subsequent tumor resections or biopsies in the Department of Neurosurgery, Huashan Hospital, from December 2020 to June 2021. Pathologic diagnoses were determined according to the 2016 World Health Organization (WHO) classification of central nervous system (CNS) tumors, and patients diagnosed with a non-glioma disease were excluded. Other exclusion criteria were the inability to cooperate with multimodal imaging examinations and pregnancy. The study protocol was approved by the Institutional Review Board of Huashan Hospital, and informed consent was provided by all participants.

### 2.2. MR Imaging Protocol

MR imaging was performed within 14 days prior to surgery. Routine clinical sequences, including contrast-enhanced T1-weighted images (TR = 6.49 ms, TE = 2.9 ms, FA = 8°, spatial resolution = 0.833 mm × 0.833 mm × 1 mm), were acquired at 3T on an Ingenia MRI scanner (Koninklijke Philips N.V., Netherlands) and MRS (PRESS; TE = 144 ms, TR = 1000 ms, spatial resolution = 13.3 mm × 13.3 mm × 15 mm) was acquired at 3T on a Discovery 750 MRI scanner (GE Healthcare, Milwaukee, USA), respectively.

CEST acquisitions were performed at 7T on an MRI scanner (MAGNETOM Terra, Siemens Healthineers, Erlangen, Germany). A prototype snapshot-CEST (optimized single-shot gradient-echo (GRE) sequence with rectangular spiral reordering) was applied (TR = 3.4 ms, TE = 1.59 ms, FA = 6°, bandwidth = 660 Hz/pixel, grappa = 3, resolution = 1.6 mm × 1.6 mm × 5 mm) [14]. Z-spectrum readings were sampled between −300 and 300 ppm, with denser sampling between −6 and 6 ppm. A total of 56 frequency offsets were obtained after saturation pulses, using three different radiofrequency (RF) power levels of B1 = 0.6, 0.75, 0.9 μT. Whole brain high-resolution T1-weighed images (MP2RAGE; TR = 3800 ms, TI1 = 800 ms, TI2 = 2700 ms, TE = 2.29 ms, FA = 7°, spatial resolution = 0.7 mm^3^ isotropic) and T2-weighed images (SPACE; TR = 4000 ms, TE = 118 ms, spatial resolution = 0.67 mm^3^ isotropic) were acquired for registration.

### 2.3. PET Imaging Protocol

The 18F-FET PET scans were performed within 14 days prior to surgery using a Biograph 128 PET/CT system (Siemens, Erlangen, Germany). Patients fasted for at least 4 h, and 370 ± 20 mBq 18F-FET was intravenously injected before scans. The uncorrected radiochemical yield was about 35%, and radiochemical purity was above 98%. The tracer was administered as an isotonic neutral solution [15]. A static scan was performed 20 min after tracer injection and lasted for 20 min. Attenuation correction was performed using low-dose CT (120 KV, 150 mA, Acq. 64 mm × 0.6 mm, 3-mm slice thickness, 0.55 pitch) before the emission scan. PET images were reconstructed using the iterative 3D method with a Gaussian filter (6 iterations, 14 subsets, full width at half maximum: 2 mm, zoom: 2) [16].

The Syngo.via workstation (Siemens, Erlangen, Germany) was used to analyze FET-PET/CT imaging. Background mean standardized uptake value (SUV) was measured in a crescent-shaped area, including gray and white matter on the contralateral hemisphere [17]. The tumor SUV_max_, SUV_peak_, SUV_mean_ and metabolic tumor volume (MTV) of lesions were semi-automatically obtained after glioma volume of interest (VOI) delineations, with a threshold of 1.6 times that of the background SUV_mean_ [18]. Background brain tissue SUV_mean_ values were determined by an experienced nuclear medicine physician, and glioma VOI delineations were transferred from MR images [19]. The maximal, peak and mean tumor-to-background ratios (TBR) were calculated as the tumor SUV_max_, SUV_peak_ and SUV_mean_ divided by the background SUV_mean_, respectively.

### 2.4. Surgery and Pathologic Evaluation

According to the 2016 WHO classification of CNS tumors (the 2021 WHO classification had not been published at the time of patient recruitment), all patients received a pathologic diagnosis from neuropathologists. Isocitrate dehydrogenase (IDH) mutation status was tested using immunohistochemistry or sequencing. For some patients, intraoperative multi-region stereotactic biopsies were also performed to validate the correlation between imaging appearance and tumor malignancy. All imaging sequences were imported into the neuro-navigation system (Medtronic S7, Minneapolis, MN, USA) and co-registered with contrast-enhanced T1-weighted images. At least three targets from the tumor core, peri-tumor region and tumor margins were linearly chosen, and the corresponding coordinates were retrieved for further analysis. After craniotomy and before a complete dural opening, cylindrical samples were obtained under conventional stereotactic biopsy procedures. After formalin fixation and paraffin embedding, the tissue sections were stained with hematoxylin and eosin (H&E) stain. Analysis of cellularity was conducted by a neuropathologist blinded to imaging analysis results. Cells were counted in four randomly selected regions of each biopsy at 400× magnification, and the average number of cell nuclei was calculated as the cell density.

### 2.5. Reproducibility of Radiomics Feature Extraction

The reproducibility assessments of the VOIs were performed by two specialists from independent segmentations of T2WI images of all patients. The inter-observer correlation coefficients were evaluated according to the Cohen k statistic (k = 0.717, Table S1). Inter-observer and intra-observer reproducibility of texture feature extractions were initially analyzed with 50 randomly chosen images from all T2 and contrast-enhanced T1-weighted images. The Cohen k statistic was conducted using SPSS 25.0 (IBM Corp. Armonk, NY, USA).

### 2.6. Imaging Analysis and Statistical Analysis

With pixel-wise fitting, APT-CEST data were calculated with the five-pool Lorentzian model using Levenberg–Marquardt algorithm [20]. The Z-spectrum data were corrected for both B0 and B1 inhomogeneities using the simultaneous mapping of water shifts and the B1 (WASABI) method [21]. Imaging modalities acquired in each were co-registered using SPM 12 software (https://www.fil.ion.ucl.ac.uk/spm/, accessed on 12 December 2021). Co-registered images were resampled into 1.6 mm × 1.6 mm × 1.5 mm, for which the voxel size is the same as for the PET images. Three regions of interest (ROIs) were defined: the MRS acquisition region, contrast-enhancing (CE) region and T2-hyperintense region. All ROIs were manually segmented by two physicians independently. Any discrepancy was resolved by consensus. For patient-wise analyses, mean CNRs, mean APT-CEST values and mean FET TBRs within the ROIs were calculated for each patient. For the voxel-wise analysis, the mean APT-CEST and mean FET TBR within each MRS voxel were calculated. Based on the corresponding ROIs, the linear relationships between APT-CEST, CNR and FET TBR were further assessed using the Pearson correlation coefficient. For the subgroup analysis, we categorized patients into (i) low-grade glioma and high-grade glioma and (ii) IDH wildtype and IDH mutant glioma. The pairwise linear comparisons between the CNR, APT-CEST and FET TBR were calculated. The CNR and APT-CEST were metrics for predicting tumor presence.

We used logistic regression to estimate the tumor probability map based on second-order polynomial features derived from MRS and APT-CEST data for each voxel. The beta coefficients were estimated by minimizing the least-squares error of the model to the tumor map classified by FET imaging. The ROC (Receiver Operating Characteristics) curve was plotted by connecting points with a coordinate of the false positive rate (1 − specificity) and the true positive rate (sensitivity) for the classifiers using various thresholds. SUV of >1.5 for the amino acid PET scan was chosen as the positive reference in ROC curve analyses. The classifier predicts positive if the data are larger than a threshold and negative if smaller than a threshold. The area under the curve (AUC) was measured as the area underneath the ROC curve, and the value varied between 0 and 1; its analysis was calculated using the method proposed by Hanley and McNeill [22]. The chi-square test was used to analyze the correlation. The strength of the correlation was assessed by the general guidelines (weak: 0.1 < |r| < 0.3; moderate: 0.3 < |r| < 0.5; strong: |r| > 0.5). A *p*-Value <0.05 was considered significant. All statistical analyses were performed using MATLAB software (R2021, Natick, MA, USA). The *p*-value of linear correlation was calculated based on the t-statistic of the hypothesis test of whether the corresponding coefficient was equal to zero or not. For group comparisons, the unpaired two-sample t-test was used.

## 3. Results

### 3.1. Patient Demographics

The patient characteristics are summarized in Table 1. Eighteen patients were enrolled in the study with three oligodendrocytomas, seven astrocytomas and eight glioblastomas. Half of the tumors were located in the temporal and insular lobes, followed by the frontal lobe (33.33%). The grade II patients were all IDH mutant, while the grade III patients were all IDH wildtype. For the WHO grade IV group, IDH mutation was detected in one patient.

### 3.2. Glioma Manifestations on Imaging 

**Figure 1:**
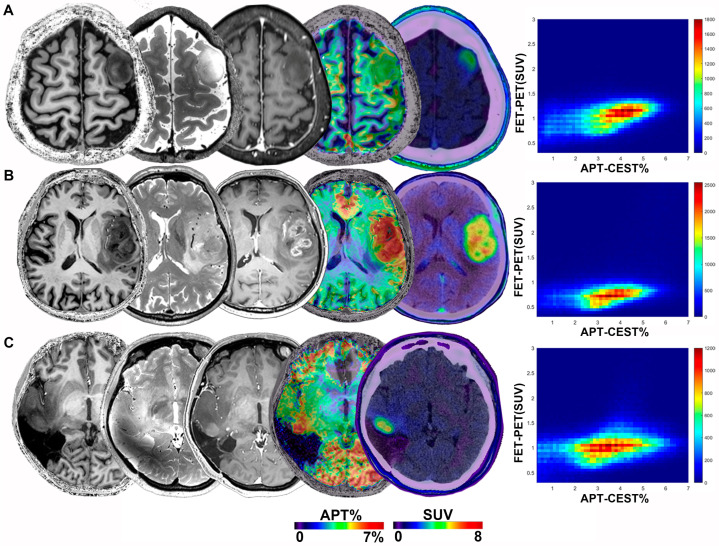
T1-weighted, T2-weighted, contrast-enhanced T1-weighted, APT-weighted MR images, FET-PET images of a 34-year-old male patient with an IDH mutant astrocytoma (WHO Grade II) (**A**), a 70-year-old male patient with an IDH wildtype glioblastoma (WHO Grade IV) (**B**) and a 46-year-old female patient with an IDH mutant recurrent astrocytoma (WHO Grade IV) (**C**). The bivariate histograms between APT values and SUV were present in the right panel, respectively.

Representative images of conventional MR, CEST and FET-PET from three patients are shown in Figure 1. The 7T APT-CEST images showed satisfactory spatial resolution and a trend of high signals in tumor areas. The average APT-CEST value in the T2 hyperintense area was highest in the IDH wildtype glioblastoma case compared with the other two (3.564% in Figure 1A, 5.560% in Figure 1B and 4.231% in Figure 1C). FET was markedly sensitive in detecting tumor occurrence and presented high SUV in all cases (1.697, 2.428 and 1.773, respectively). Both high FET and APT-CEST areas were grossly more extensive than contrast-enhancing areas (Figure 1B), indicating tumor infiltration in the intact BBB region.

**Figure 2:**
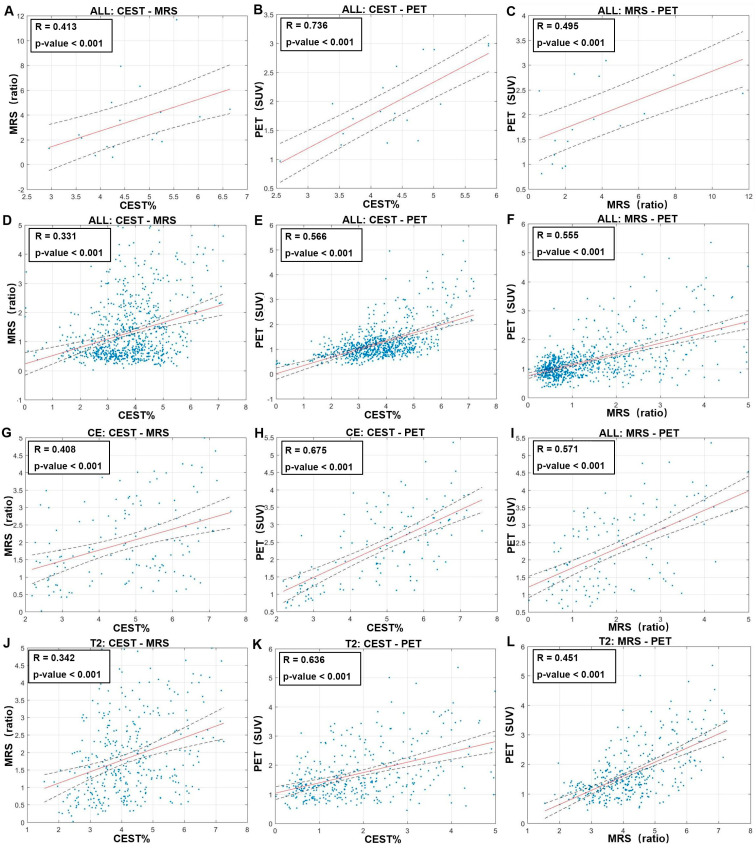
Correlations between APT-CEST, MRS and FET-PET. Patient-wise correlations of CEST−MRS (**A**), CEST−FET (**B**) and MRS−FET (**C**) in all regions; voxel-wise correlations of CEST−MRS (**D**), CEST−FET (**E**) and MRS−FET (**F**) in all regions. Correlations of CEST−MRS (**G**), CEST−FET (H) and MRS−FET (**I**) in voxels of contrast-enhanced regions; correlations of CEST−MRS (**J**), CEST−FET (**K**) and MRS−FET (**L**) in voxels of T2−hyperintense regions.

The average APT, CNR and SUVs of each patient enrolled in the study were calculated and compared. The patient-wise analysis (Figure 2A–C) revealed a strong CEST-FET correlation (R = 0.736, *p* < 0.001), a moderate MRS-FET correlation (R = 0.495, *p* = 0.037) and no significant CEST-MRS correlation. The voxel-wise analysis (Figure 2D–F) showed that FET was strongly correlated with both CEST (R = 0.566, *p* < 0.001) and MRS (R = 0.555, *p* < 0.001), whereas correlation of CEST-MRS is moderate (R = 0.331, *p* < 0.001). Subgroup analysis was further performed based on contrast-enhancing (Figure 2G–I) and T2-hyperintense areas (Figure 2J–L). Both subgroups had increased CEST and FET correlation coefficients (contrast-enhancing: R = 0.675, *p* < 0.001; T2-hyperintense: R = 0.636, *p* < 0.001), while the CEST-MRS correlations were slightly improved in both (contrast-enhancing: R = 0.408, *p* < 0.001; T2-hyperintense: R = 0.342, *p* < 0.001). The MRS-FET correlation was stronger in contrast-enhancing area (R = 0.571, *p* < 0.001) but weaker in T2-hyperintense area (R = 0.451, *p* < 0.001). In summary, both CEST and MRS were correlated with FET-PET, while the correlation between the two MR sequences was weaker, indicating different imaging mechanisms and potential complementarity.

### 3.3. Tumor Grade and IDH Status 

**Figure 3:**
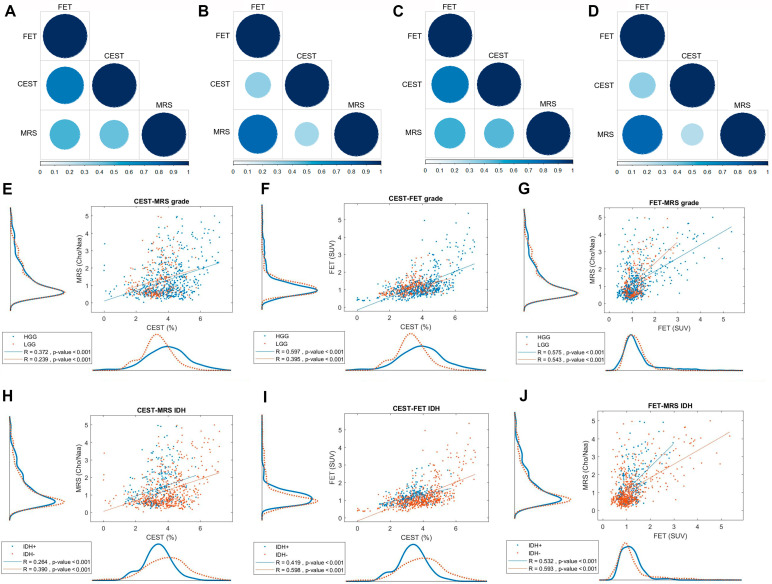
Correlations between APT-CEST, MRS and FET-PET according to tumor grade and IDH status. Patient−wise correlation plots depicting in high-grade glioma (**A**), low grade glioma (**B**), IDH wildtype group (**C**) and IDH mutant group (**D**). The size and color depth of the plots are proportional to the correlation, and correlation index from 0 to 1 was shown below; (**E**−**G**) Voxel−wise correlations according to tumor grade, CEST and MRS (**E**), CEST and FET PET(**F**) and FET and MRS (**G**). (**H**−**J**) Voxel−wise correlations according to the IDH status, CEST and MRS (**H**), CEST and FET PET(**I**) and FET and MRS (**J**).

Categorized by tumor grade, the subgroup analysis of the correlation between different imaging modalities was conducted. In high-grade glioma (HGG) patients, CEST demonstrated a strong correlation with FET (R = 0.765, *p* < 0.001), while both CEST-MRS (R = 0.426, *p* = 0.128) and MRS-FET (R = 0.475, *p* = 0.086) correlations showed moderate trends toward statistical significance (Figure 3A). In low-grade glioma (LGG) patients, no obvious correlations were found among the three modalities (Figure 3B). IDH mutation, which predicts temozolomide efficacy and patient survival, is currently the key molecular marker for glioma [23]. Subgroup analysis of IDH status found CEST was strongly correlated with FET (R = 0.758, *p* = 0.003, Figure 3C), while no significant correlation was found between the imaging modalities in the IDH mutant group, although a positive trend was seen between MRS and FET (Figure 3D).

Considering that limited sample size might lead to these negative results, we furtherly performed the voxel-wise analysis on tumor grade and IDH status, which indicated that CEST-MRS (Figure 3E) and CEST-FET (Figure 3F) correlations in the HGG group remained superior to those in LGG group, while a strong correlation between MRS and FET (Figure 3G) in both subgroups (*p* < 0.001). Moreover, CEST histogram analysis revealed that the HGG peak was distinct from the LGG peak. Together, these data implied a role for the imaging modalities in differentiating tumor grade and IDH status, which was especially true for CEST. However, the CEST-FET correlation was stronger in the IDH wildtype group than in the mutant group (Figure 3I), and a strong correlation between MRS and FET regardless of IDH mutation (*p* < 0.001, Figure 3J); while the CEST-MRS correlations were moderate and weak (Figure 3H), respectively. Similarly, histogram distributions for CEST were different between IDH mutant and wildtype groups.

Therefore, the mean APT, CNR and SUV values extracted from all voxels were compared according to tumor grades and IDH status, confirming that APT values were significantly higher in HGG and IDH wildtype group (tumor grade: 3.923 ± 1.239 vs. 3.317 ± 0.868, *p* < 0.001; IDH status: 3.932 ± 1.264 vs. 3.358 ± 0.847, *p* < 0.001). In contrast, no significant difference in CNR was detected (Table 2).

The tumor grade and IDH status indicated that CEST-MRS and CEST-FET correlations in the HGG group remained superior to those in the LGG group, while a strong MRS-FET correlation in both subgroups (*p* < 0.001). Higher correlations can be found in IDH wildtype groups than in mutation groups. Note that both the FET and MRS value distributions of high and low tumor grades or IDH mutate and IDH wildtype overlapped, and it is difficult to distinguish the tumor grade or IDH status based on the distribution. However, the APT-CEST value distribution of high and low tumor grades or IDH mutate and IDH wildtype clearly peaked at different values.

### 3.4. Diagnostic Accuracy 

**Figure 4:**
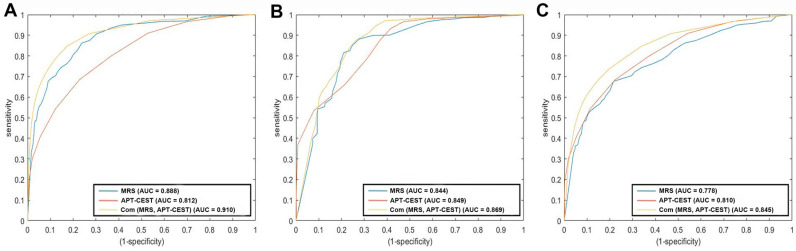
Voxel−wise predictions of high FET-PET signals using APT−CEST, MRS and a combination of the methods. (**A**) ROC curves in all voxels. (**B**) ROC curves of the voxels in the contrast-enhancing regions. (**C**) ROC curves of the voxels in the T2−hyperintense regions. The x− and *y*−axis of the ROC (Receiver Operating Characteristics) curve were the false positive rate (FPR is known as (1 − specificity)) and the true positive rate (TPR is known as sensitivity), respectively. SUV of >1.5 for the amino acid PET scan was chosen as the positive reference in ROC curve analyses. The area under the curve (AUC) and its analysis was calculated using the method proposed by Hanley and McNeill. AUC measures the area underneath the ROC curve and varies between 0 and 1.

By using FET-PET as the relative standard for predicting tumor presence, we constructed ROC curves to test the diagnostic accuracies of CEST and MRS (Supplemental Tables S2 and S3). Voxel-wise analysis showed that the AUCs of CEST and MRS were 0.812 (95% CI: 0.808–0.815) and 0.888 (95% CI: 0.885–0.891) (Figure 4A), respectively. When the two methods were combined, the AUC increased to 0.910 (95% CI: 0.907–0.913). For the voxel subgroups in the contrast-enhancing areas, the diagnostic accuracy of CEST and MRS were similar, with an AUC of 0.849 (95% CI: 0.842–0.856) and 0.844 (95% CI: 0.837–0.852), respectively, and increased to an AUC of 0.869 (95% CI: 0.862–0.875) when combined (Figure 4B). In T2-hyperintense areas, CEST was superior to MRS, with AUCs of 0.810 (95% CI: 0.805–0.814) and 0.778 (95% CI: 0.773–0.783), respectively. Their combination also improved the diagnostic accuracy with an AUC of 0.845 (95% CI: 0.841–0.849) (Figure 4C).

**Figure 5:**
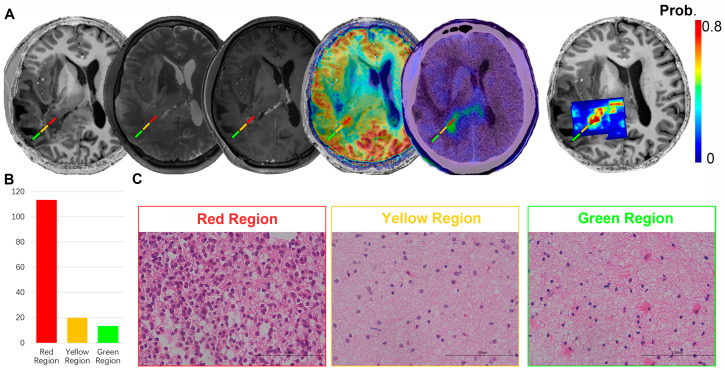
Probability maps of the tumor presence using APT-CEST/MRS combination. (**A**) T1-weighted, T2-weighted, contrast-enhanced T1-weighted, APT-weighted MR images, FET-PET images, and the probability mapping of tumors (possibility from 0 to 0.8) in a 41-year-old male with an IDH2 mutant oligodendroglioma (WHO Grade II). (**B**) Cell density counts were performed in 3 biopsy regions. (**C**) The histopathologic results of 3 biopsies (H&E stain, ×400 magnification). Red region, yellow region and green region stand for tumor core zone, perilesional zone and normal zone, relatively.

We then combined CEST and MRS data to develop probability maps of tumor presence (Supplementary Figure S1). An exemplary case with three stereotactic biopsies marked with different colors is shown in Figure 5. The red region represented the tumor core according to contrast-enhanced T1 imaging, where the map indicated high-signal intensity with a tumor prediction probability of 64.50%. H&E staining of the biopsy tissue confirmed atypical nuclear accumulation with a cell density of 113.25, indicating that the tumor had high proliferative activity. The yellow region was targeted 2 cm beyond the contrast-enhancing area, which is usually considered the peri-tumoral zone. The resultant map showed a tumor prediction probability of 14.00%, which was further confirmed by H&E staining showing tumor presence with a cell density of 20.00 and mild atypical nuclear distributions. For the peripheral green region, the map predicted that no tumor was present. The H&E staining revealed scattered atypical nuclei with the lowest cell density at 13.50 (Supplemental Table S4). In summary, the probability map showed an optimal efficacy in the ability to predict tumor presence.

## 4. Discussion

Precise delineation of glioma boundaries guides the extent of tumor resection and irradiation, thus having a potential impact on patient survival. PET scan, which traces metabolites in vivo to reflect tumor malignancy, is the most recommended technique for evaluating glioma boundaries. In this study, we tried to find a noninvasive MR alternative by developing novel sequences, introducing ultra-high-field imaging and combining multiple modalities. CEST MRI benefits from ultra-high field (UHF) strengths due to higher signal–noise ratio (SNR) and higher chemical shift separation. Thus, some amine protons with low concentration, which may not be visible at 3T by the CEST methodology, could be probed at 7T. The results revealed a complementary relationship between CEST and MRS in evaluating local metabolic characteristics. To our knowledge, this is the first report implementing ultra-high field CEST/MRS multimodal imaging in delineating glioma metabolic boundaries.

Our analysis revealed that APT values in CEST and CNR values in MRS were strongly correlated with FET values at the voxel-wise level, while the correlation between the two MR sequences was weaker. This result confirmed that CEST and MRS could evaluate tumor metabolic activity, yet via different mechanisms. The APT-CEST chooses 3.5 ppm radiofrequency pulses to detect the amide protons of endogenous peptides and mobile proteins. Since the concentration of peptides and mobile proteins was positively correlated with tumor proliferation and significantly elevated in high-grade brain tumors [24], APT imaging might indirectly reflect tumor malignancy and was exploited to differentially diagnose glioma [25], predict IDH mutation [26] and differentiate tumor progression from radio-necrosis [10]. Comparatively, MRS directly quantifies water-soluble metabolite concentrations according to their precession frequency. By eliminating confounders such as baseline variation, the CNR can reflect local tumor malignancy and has been used to differentiate glioma grades and navigate resections, especially in areas beyond the contrast signals.

Glioma grades are classified as I–IV according to WHO and are further subtyped based on molecular characteristics such as IDH mutation [27]. Preoperative prediction of such information plays an important role in designing surgical strategies and deciding the extent of resection. Conventional T1-contrast imaging has been used to differentiate HGG from LGG according to the appearance of contrast signals, while the T2-FLAIR mismatch sign has been identified to predict IDH mutation [28,29]. Aside from the conventional approaches, previous studies used CEST to predict HGG and IDH mutations with an AUC of 0.84 and 0.89 [10,30], respectively. By identifying the 2-HG peak on edited MRS analysis, the AUC for predicting IDH mutations was reported as 0.84 [31]. Although only 18 patients were enrolled, we also revealed that the APT and CNR values of HGG patients were higher than LGG ones, and IDH wildtype cases generated higher APT values than mutant cases, which is consistent with previous research. Interestingly, our results showed that the correlations among CEST, MRS and FET-PET were similar between HGG and LGG subgroups, indicating that such imaging approaches are consistent in the ability to trace tumor metabolism regardless of independent prognostic factors such as tumor grade.

Multimodal imaging has emerged as a general trend for clinical glioma theragnostic since it is impossible for a single imaging modality such as CEST, which evaluates amino acid metabolism rather than local blood supplies or cell densities, to assess tumor characteristics thoroughly. In this study, we used voxel-wise FET-PET as a reference and demonstrated that the CEST/MRS combination was superior to a single modality in indicating glioma presence.

Gliomas grow along white matter fiber tracts in an infiltrative manner, and clinical prognoses are positively correlated with the extent of tumor removal. However, for tumors located in eloquent regions, extensive resection always leads to functional impairment. Thus, precise identification of tumor boundaries is key in the surgical management of glioma. Studies found that gliomas usually recurred within a 2 cm margin from the contrast-enhancing area [15], indicating that glioma infiltrates beyond the boundary delineated by conventional MR imaging. FET-PET has been recommended by the latest EANO guidelines as a superior technique for delineating glioma extent, but low spatial resolution limits its detailed guidance for tumor resection. Although MRS was also reported to navigate glioma metabolic boundaries, this technique is routinely present as a single slice and insufficient for delineating tumors in three dimensions [32]. In contrast, CEST has been reported to possibly indicate glioma infiltration, and ultra-high fields markedly increase its spatial resolution [33,34]. Our results revealed a significant correlation between CEST and FET-PET signals and showed that its combination with MRS could predict glioma presence outside the contrast-enhancing area.

In this study, the *p*-value for the correlation coefficients between voxel-wise APT-CEST, MRS and FET-PET data is very low, and this is because we used large samples in the statistical analysis. If the actual correlation between two data is not zero, the *p*-value will be smaller when a larger number of samples is used [35]. It is also important to clarify the influence of data dependency between voxels. In the voxel-wise analysis, we used a large voxel size, 13.3 mm × 13.3 mm × 15 mm, such that CEST, MRS and FET-PET imaging methods can provide good spatially independent information between adjacent voxels. No clustered data points in Figure 2 confirmed that the data dependency between voxels is small.

There were some limitations in this study. First, considering that the latest WHO 2021 CNS tumor classification had not been published during the study, diagnoses given by pathologists were based on the former edition (WHO 2016 CNS classification), which has been adjusted in our undergoing studies. Second, FET-PET was chosen as the reference to detect tumor metabolism, while tumor biopsies were only used for direct comparison in one case during the preliminary validation. A prospective clinical trial (ICTRP Registration No. ChiCTR2000036816) was registered to further identify the pathologic margins of the lesion; a biopsy-proven “golden” reference will be introduced in the further study. In the future, we will use our model to guide surgery, such as integrating tumor probability maps into navigation machines to guide surgical resection in real time. Further analysis of multimodal signals with multiple intra-operative biopsies was designed to validate and reach the goal of gross total resection. Third, the sample size was small, lowering statistical power when the patient-wise analysis was performed to identify unique manifestations in each subgroup. We are still recruiting patients in our undergoing study, and in the future, AI (artificial intelligence) will be introduced to generate a real-time tumor delineation mapping based on our intra-operative MRI to realize the extreme balance between tumor resection and function reservation. Forth, different modalities were co-registered manually, causing inevitable spatial incongruence. PET-MRI might improve this problem but will not be widely applied in the near future. Additionally, in this article, we mainly focused on preoperative prediction for more precise glioma delineation, optimizing tumor resection and irradiation guidance; multi-factor analysis, such as the other radiological parameters that impact prognosis such as multifocal or paraventricular lesions [36], was not involved in this article.

## 5. Conclusions

CEST and MRS were positively associated with FET-PET and predicted active tumor metabolism with high diagnostic accuracy. Such advanced MR imaging combinations under ultra-high fields could pave the way for more precise glioma delineation beyond conventional contrast-enhancing or T2-hyperintense boundaries, thus optimizing tumor resection and irradiation guidance.

## Figures and Tables

**Table 1:** Patient demographics.

		All Patients	WHO II	WHO III	WHO IV
No. of patients					
		18	4	5	9
Age (yrs)					
	Mean	49.5	43	40.4	57.4
	Range	28–75	32–65	28–47	36–75
Gender					
	Male	11 (61.11%)	2 (50%)	2 (40%)	7 (77.78%)
	Female	7 (38.89%)	2 (50%)	3 (60%)	2 (22.23%)
Position					
	Frontal Lobe	6 (33.33%)	2 (50%)	2 (40%)	2 (22.22%)
	Parietal Lobe	1 (5.56%)	1 (25%)	0	0
	Occipital Lobe	0	0	0	0
	Temporal and Insular Lobe	9 (50%)	1 (25%)	3 (60%)	5 (55.56%)
	Others	2 (11.11%)	0	0	2 (22.22%)
IDH status					
	Wildtype	13 (72.22%)	0	5 (100%)	8 (88.89%)
	Mutant	5 (27.78%)	4 (100%)	0	1 (11.11%)

**Table 2:** Pixel-wise comparison of APT-CEST, MRS and FET-PET according to tumor grade and IDH mutation.

		Mean	Std	*p*-Value
**APT-CEST (%)**	HGG	3.923	1.239	<0.001
	LGG	3.317	0.868	
	IDH mutant	3.358	0.846	<0.001
	IDH wildtype	3.932	1.264	
**FET-PET (SUV)**	HGG	1.272	0.763	0.037
	LGG	1.161	0.422	
	IDH mutant	1.184	0.412	0.115
	IDH wildtype	1.266	0.780	
**MRS (CNR)**	HGG	1.295	1.023	0.889
	LGG	1.284	0.967	
	IDH mutant	1.360	1.012	0.183
	IDH wildtype	1.258	1.002	

HGG: high-grade glioma. LGG: low-grade glioma. SUV: standardized uptake value. CNR: Cho/NAA ratio. Std: Standard deviation.

## Data Availability

The raw data that support the findings of this study are available from the corresponding author, Qi Yue and Ying Mao, upon valid request.

## Supplementary Materials

The following supporting information can be downloaded at: https://www.mdpi.com/article/10.3390/metabo12100901/s1, Figure S1: ROC curve combining CEST and MRS to predict high FET SUV for the example shown in Figure 5, Table S1: Analyses of interobserver agreement; Table S2: Regression model for combination of CEST and MRS; Table S3: Parameters of the ROC curves in Figure 4; Table S4: The values of CEST, MRS, FET and tumor probabilities of the three biopsy regions in Figure 5.
