# Supplementary material for: Noninvasive Delineation of Glioma Infiltration with Combined 7T Chemical Exchange Saturation Transfer Imaging and MR Spectroscopy: A Diagnostic Accuracy Study
Source: Metabolites. 2022 Sep 24;12(10):901. doi: 10.3390/metabo12100901 (PMC9607140; doi:10.3390/metabo12100901)
Supplement: Supplementary file 1 [file metabolites-12-00901-s001.zip › Table S1.pdf]

Supplemental Table S1. Analyses of interobserver agreement. (k < 0, poor agreement; k = 0–0.20, slight agreement; k = 0.21–0.40, fair agreement; k = 0.41–0.60, moderate agreement; k = 0.61–0.80, substantial agreement; k = 0.81–1.00, almost perfect agreement)

		Neurosurgeon		Total
		**Normal Brain**	**Tumor**	
**Radiologist**	**Normal Brain**	24	3	27
	**Tumor**	4	19	23
**Total**		28	22	50
