# Supplementary material for: Noninvasive Delineation of Glioma Infiltration with Combined 7T Chemical Exchange Saturation Transfer Imaging and MR Spectroscopy: A Diagnostic Accuracy Study
Source: Metabolites. 2022 Sep 24;12(10):901. doi: 10.3390/metabo12100901 (PMC9607140; doi:10.3390/metabo12100901)
Supplement: Supplementary file 1 [file metabolites-12-00901-s001.zip › Table S2.pdf]

Supplemental Table S2. Regression model for combination of CEST and MRS.

ANOVA: CEST and MRS

	df	SS	MS	F	p-value
**Regression**	2	168.237	84.119	344.132	<0.001
**Residual**	769	187.972	0.244		
**Total**	771	356.209			

ANOVA: MRS

	df	SS	MS	F	p-value
**Regression**	1	109.872	109.872	343.436	<0.001
**Residual**	770	246.337	0.320		
**Total**	771	356.209			

Add CEST as a new variable can improve model

	dfE	SSE	MSE	F	p-value
**Full model (CEST and MRS)**	769	187.972	0.244	238.776	<0.001
**Single variable model (MRS)**	770	246.337			

ANOVA: Analysis of Variance. df: degree of freedom. SS: Sum of Squares. MS: Mean Square. dfE: degree of freedom error. SSE: Sum of Squares due to Error. MSE: Mean Square Error
