# Supplementary material for: Noninvasive Delineation of Glioma Infiltration with Combined 7T Chemical Exchange Saturation Transfer Imaging and MR Spectroscopy: A Diagnostic Accuracy Study
Source: Metabolites. 2022 Sep 24;12(10):901. doi: 10.3390/metabo12100901 (PMC9607140; doi:10.3390/metabo12100901)
Supplement: Supplementary file 1 [file metabolites-12-00901-s001.zip › Table S3.pdf]

Supplemental Table S3. Parameters of the ROC curves in Figure 4.

		AUC	95%C.I.		p-value
	**MRS**	0.888	0.885	0.891	<0.001
**A**	**APT-CEST**	0.812	0.808	0.814	<0.001
	**Com (MRS, APT-CEST)**	0.910	0.907	0.913	<0.001
	**MRS**	0.778	0.773	0.783	<0.001
**B**	**APT-CEST**	0.810	0.805	0.814	<0.001
	**Com (MRS, APT-CEST)**	0.845	0.841	0.849	<0.001
	**MRS**	0.844	0.837	0.852	<0.001
**C**	**APT-CEST**	0.849	0.842	0.856	<0.001
	**Com (MRS, APT-CEST)**	0.869	0.862	0.875	<0.001

Com: combine. AUC: Area under curve. C.I.: confidence interval.
