# Supplementary material for: Noninvasive Delineation of Glioma Infiltration with Combined 7T Chemical Exchange Saturation Transfer Imaging and MR Spectroscopy: A Diagnostic Accuracy Study
Source: Metabolites. 2022 Sep 24;12(10):901. doi: 10.3390/metabo12100901 (PMC9607140; doi:10.3390/metabo12100901)
Supplement: Supplementary file 1 [file metabolites-12-00901-s001.zip › Table S4.pdf]

Supplemental Table S4. The values of CEST, MRS, FET and tumor probabilities of the three biopsy regions in Figure 5.

	**Red Region**		**Yellow Region**		**Green Region**	
	**Median**	**Std**	**Median**	**Std**	**Median**	**Std**
**Model (%)**	64.500	15.536	14.000	5.758	0.000	3.024
**CEST (%)**	4.830	0.407	3.880	0.412	4.230	0.248
**FET (SUV)**	3.580	0.089	1.625	0.642	0.770	0.083
**MRS (CNR)**	3.770	0.598	2.280	0.797	0.000	0.321

Std: standard deviation.
