# Supplementary figures and images for: Noninvasive Delineation of Glioma Infiltration with Combined 7T Chemical Exchange Saturation Transfer Imaging and MR Spectroscopy: A Diagnostic Accuracy Study
Source: Metabolites. 2022 Sep 24;12(10):901. doi: 10.3390/metabo12100901 (PMC9607140; doi:10.3390/metabo12100901)

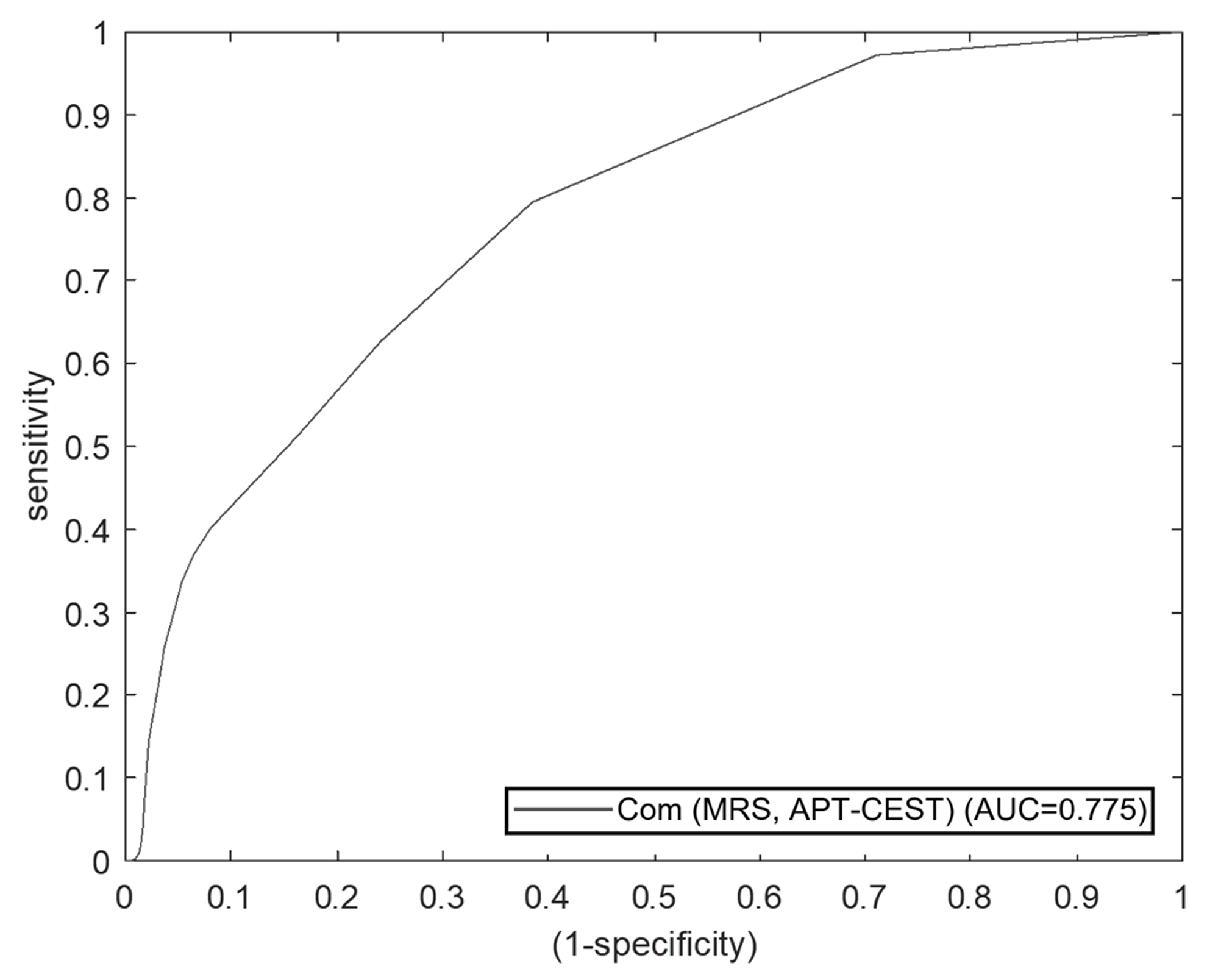

Supplement: Supplementary file 1 [file metabolites-12-00901-s001.zip › Figure S1.tif]
